# Metagenomic gut microbiome analysis of Japanese patients with multiple chemical sensitivity/idiopathic environmental intolerance

**DOI:** 10.1186/s12866-024-03239-y

**Published:** 2024-03-11

**Authors:** Kentaro Watai, Wataru Suda, Rina Kurokawa, Kiyoshi Sekiya, Hiroaki Hayashi, Maki Iwata, Kisako Nagayama, Yuto Nakamura, Yuto Hamada, Yosuke Kamide, Yuma Fukutomi, Takeru Nakabayashi, Kosei Tanaka, Masahiro Kamita, Masami Taniguchi, Masahira Hattori

**Affiliations:** 1 Center for Immunology and Allergy, Shonan Kamakura General Hospital, 1370-1 Okamoto, Kamakura, Kanagawa 247-8533 Japan; 2 Clinical Research Center for Allergy and Rheumatology, NHO Sagamihara National Hospital, Sagamihara, Kanagawa Japan; 3 Laboratory for Microbiome Sciences, RIKEN Center for Integrative Medical Sciences, Yokohama, Kanagawa Japan; 4 Graduate School of Advanced Science and Engineering, Waseda University, Tokyo, Japan; 5 H.U. Group Research Institute G.K., Akiruno, Tokyo, Japan

**Keywords:** Multiple chemical sensitivity, Central nervous system, Gut microbiome, Shotgun metagenomic sequencing

## Abstract

**Background:**

Although the pathology of multiple chemical sensitivity (MCS) is unknown, the central nervous system is reportedly involved. The gut microbiota is important in modifying central nervous system diseases. However, the relationship　between the gut microbiota and MCS remains unclear. This study aimed to identify gut microbiota variations associated with MCS using shotgun metagenomic sequencing of fecal samples.

**Methods:**

We prospectively recruited 30 consecutive Japanese female patients with MCS and analyzed their gut microbiomes using shotgun metagenomic sequencing. The data were compared with metagenomic data obtained from 24 age- and sex-matched Japanese healthy controls (HC).

**Results:**

We observed no significant difference in alpha and beta diversity of the gut microbiota between the MCS patients and HC. Focusing on the important changes in the literatures, at the genus level, *Streptococcus*, *Veillonella*, and *Akkermansia* were significantly more abundant in MCS patients than in HC (*p* < 0.01, *p* < 0.01, *p* = 0.01, respectively, fold change = 4.03, 1.53, 2.86, respectively). At the species level, *Akkermansia muciniphila* was significantly more abundant (*p* = 0.02, fold change = 3.3) and *Faecalibacterium prausnitzii* significantly less abundant in MCS patients than in HC (*p* = 0.03, fold change = 0.53). Functional analysis revealed that xylene and dioxin degradation pathways were significantly enriched (*p* < 0.01, *p* = 0.01, respectively, fold change = 1.54, 1.46, respectively), whereas pathways involved in amino acid metabolism and synthesis were significantly depleted in MCS (*p* < 0.01, fold change = 0.96). Pathways related to antimicrobial resistance, including the two-component system and cationic antimicrobial peptide resistance, were also significantly enriched in MCS (*p* < 0.01, *p* < 0.01, respectively, fold change = 1.1, 1.2, respectively).

**Conclusions:**

The gut microbiota of patients with MCS shows dysbiosis and alterations in bacterial functions related to exogenous chemicals and amino acid metabolism and synthesis. These findings may contribute to the further development of treatment for MCS.

**Trial registration:**

This study was registered with the University Hospital Medical Information Clinical Trials Registry as UMIN000031031. The date of first trial registration: 28/01/2018.

**Supplementary Information:**

The online version contains supplementary material available at 10.1186/s12866-024-03239-y.

## Background

Multiple chemical sensitivity (MCS) is a disease with multi-organ manifestations caused by trace amounts of nonspecific chemicals and environmental factors. Symptoms may be induced by environmental factors other than chemical substances, a phenomenon termed “idiopathic environmental intolerance” by the World Health Organization in 1996 [1, 2].

Although the pathology of MCS is unknown, “central sensitization” has been suggested as a disease mechanism of MCS. Central sensitization is a condition in which the central nervous system is overexcited by chronic stimulation from the peripheral nerves, i.e., signals from the peripheral nerves to the central nervous system are not inhibited and amplified centrally [3]. The brain and gut environment, including the gut microbiota, are closely related via the autonomic nervous system and humoral factors (hormones, cytokines, short-chain fatty acids etc.). This bidirectional relationship is referred to as the "gut–brain axis” [4]. The association between the central nervous system and the gut microbiota has been studied in some diseases, such as multiple sclerosis and autism [5, 6]. Some patients with MCS are comorbid with irritable bowel syndrome and clinically complain of gastrointestinal symptoms [7]. However, there is no report on gut microbiome analysis in MCS.

In a previous study, we clarified the relationship between MCS and birth by caesarean section [8]. Neonates born by cesarean section have alterations in their gut microbiota due to a lack of exposure to the microbiota in the birth canal. These gut microbiota alterations influence certain central nervous system disorders via gut–brain interaction [9]. In this study, we aimed to identify gut microbiota variations associated with MCS using shotgun metagenomic sequencing of fecal samples.

## Methods

### Study design

This was a prospective study of Japanese patients with MCS performed between February 2018 and March 2018. Thirty consecutive Japanese female patients with MCS visiting Sagamihara National Hospital were included in the study. Inclusion criterion were: 1) aged 19 or more, 2) female patients with MCS. Exclusion criterion were 1) receiving treatment with antibiotics and/or proton pump inhibitors (PPIs) within the last six months, 2) BMI ≥ 30 kg/m^2^, 3) having inflammatory bowel disease, type 2 diabetes, liver cirrhosis, or colorectal cancer. The exclusion criteria were intended to avoid including gut microbiota alterations due to factors other than MCS [10–17].

Fecal DNA metagenomic sequencing data of 104 healthy controls (HC) were obtained from research conducted in Japan between 2010 and 2013 [18]. From these data, data from 24 age- and sex-matched female HC were selected for comparison with the cases in this study.

The ethics committee of the National Hospital Organization approved the study protocol (No. 27 in 2017). The study participants’ informed consents were obtained when they were registered. This study was registered with the University Hospital Medical Information Clinical Trials Registry as UMIN000031031 (The date of first trial registration: 28/01/2018).

### Fecal sample collection

Fresh feces were collected and stored under anaerobic conditions in an AnaeroPack™ (Mitsubishi Gas Chemical Co. Inc., Tokyo, Japan) at 4 °C. Within 36 h of sample collection, the feces were frozen in liquid nitrogen and stored at –80 °C until analysis.

### Fecal DNA isolation and metagenomic sequencing

Fecal DNA samples were prepared as described previously [19]. In brief, DNA was isolated from the feces with an enzymatic lysis method using lysozyme (Sigma-Aldrich Co. Llc., Tokyo, Japan) and achromopeptidase (FUJIFILM Wako Pure Chemical Corporation, Osaka, Japan). The DNA was purified by treatment with RNase A (FUJIFILM Wako Pure Chemical Corporation), followed by precipitation with a 20% PEG solution (PEG6000 in 2.5 M NaCl). The DNA was pelleted by centrifugation, rinsed with 75% ethanol, and dissolved in TE buffer. The fecal DNA samples were sequenced using the MiSeq (Illumina, Inc. San Diego, CA, USA) sequencing system according to the manufacturer’s instructions. In brief, after quality filtering, the reads were mapped to a human genome (hg19) and phiX bacteriophage genomes were removed. The high-quality reads were used for further analysis.

### Mapping of the metagenomic reads to reference genomes

For microbial genome/species assignment of the metagenomic reads, 500,000 high-quality metagenomic reads per individual were mapped to reference genomes using Bowtie2 (v2.2.1), with a 95% identity threshold, as described previously [18]. To improve the efficiency and accuracy of taxonomic assignment of the metagenomic sequences and reduce excess computing loads, we used an in-house developed reference genome database including 2,788 complete and 22,317 draft genomes available from GenBank/EBI/DDBJ, comprising a total of 6,149 genomes representing 2,373 clusters at the species level of Bacteria and Archaea [18]. The number of multi-hit reads that mapped to multiple genomes with identical scores was normalized by the proportion to the number of reads uniquely mapped to the genomes. The relative abundance of each genome was calculated by normalizing the number of reads mapped to the genome by the total number of reads mapped. NCBI taxonomy information was used for taxonomic assignment of phylum, genus, and species for each genome. Genomes that were not assigned to a particular taxonomic rank were assigned to the higher rank classification and designated “unclassified higher rank.”

### Assembly of metagenomic sequences and gene prediction

For each individual, the filter-passed MiSeq reads were assembled using MEGAHIT (v1.2.4). Prodigal (v2.6.3) was used to predict protein-coding genes (≥ 100 bp) in contigs (≥ 500 bp) and singletons (≥ 300 bp). Finally, 6,150,821 non-redundant genes were identified in the 30 MCS samples by clustering the predicted genes using CD-HIT [20] with a 95% nucleotide identity and 90% length coverage cut-off. The Good's coverage index of the data used was 0.79, indicating that most genes were covered [21].

### Functional assignment of non-redundant genes

The non-redundant genes were functionally assigned by alignments against the Kyoto Encyclopedia of Genes and Genomes (KEGG) database (release 2019–10-07) using DIAMOND (e-value ≤ 1.0e − 5) [22] to obtain KEGG orthologies (KOs). Genes with a best hit to eukaryotic genes were excluded from further analysis.

### Quantification of the annotated genes

Per individual, 500,000 metagenomic reads were mapped to the Japanese gut microbiome and integrated gene catalog merged reference gene set [18, 23] using Bowtie2 with a 95% identity cut-off. The number of reads that mapped equally to multiple genes was normalized by the proportion of the number of reads uniquely mapped to the genes. The proportions of KOs were calculated from the number of reads mapped to them. Wilcoxon’s rank sum tests were used to determine the statistical significance of differences between the two groups.

### Assessment and selective criteria of MCS (cases)

The most widely used instrument for evaluating MCS in adult populations is the Quick Environmental Exposure and Sensitivity Inventory (QEESI), a validated questionnaire that is both sensitive (92%) and specific (95%) for MCS [24–28]. Researchers from various countries, including the United States, Japan, and Germany, have used the QEESI to assess MCS. The Japanese version of QEESI has been validated [24]. To strictly select patients with MCS, the QEESI as well as the physician’s diagnosis was used. The QEESI consists of five sections: I Chemical Exposures, II Other exposures, III Symptoms, IV Masking Index, and V Impact of sensitivities. Each section other than Masking Index is scored on a 0–100 scale. Moreover, a risk criteria classification is included in I Chemical Exposures, III Symptoms, and IV Masking Index. A section I total score ≥ 40 and section III total score ≥ 40 was defined as “very suggestive” and these scores were used as cut-offs to select patients with MCS. For more detail, refer to the Supplementary Materials in Additional file 1.

### Statistical analysis

Demographic variables, such as the age and body mass index, were analyzed using t-tests or Mann–Whitney U tests based on normality test using SPSS v.21.0 software (IBM Corp, Armonk, NY, USA). Specifically, the t-test was used to compare age, and the Mann–Whitney U test was used to compare BMI. For the overall comparison of microbial compositions between MCS and HC, alpha diversity was evaluated based on the number of species and the Shannon index for each sample group. The results were analyzed using Mann–Whitney U tests. Beta diversity was evaluated using principal coordinate (PCo) analysis based on the Bray–Curtis distance. Permutational multivariate analysis of variance (PERMANOVA) was used to assess the associations of age and BMI with the gut microbiota structure using the adonis function in the Vegan package in R. Differences in the abundance of each phylum/genus/species and functional pathways of the gut microbiome between MCS and HC were analyzed using Mann–Whitney U tests. A *p* value < 0.05 was considered statistically significant.

## Results

### Study participant characteristics

We recruited 30 female patients with MCS (mean age, 48.1 ± 11.6 years). Metagenomic gut microbiome data of the patients with MCS were newly collected in this study and compared with published metagenomic data of gut microbiomes from 24 age- and sex-matched HC (mean age 41.7 ± 12.8 years; Table 1).

**Table 1:** Patient characteristics

	**HC**	**MCS**	***p*-value**
n	24	30	—
Sex (male/female)	0/24	0/30	—
Age (years) mean ± SD	41.7 ± 12.8	48.1 ± 11.6	0.06
Body mass index, median (IQR)	21.5 (20.6–22.9)	22.6 (21.8–23.3)	0.17
Disease duration (years), median (IQR)	—	1.75 (1.08–2.77)	—

*HC* healthy controls, *MCS* multiple chemical sensitivity

### Differences in overall gut microbiota diversity between MCS patients and HC

**Fig. 1:**
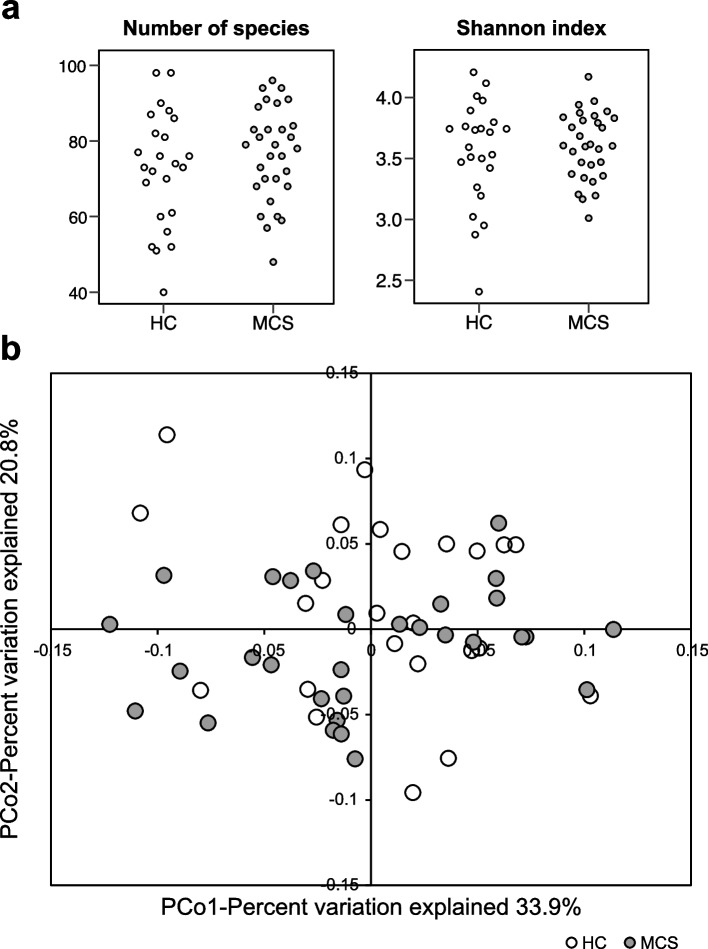
Alpha and beta diversity in patients with multiple chemical sensitivity (MCS, *n* = 30) and healthy controls (HC, *n* = 24). **a** Dot plots showing alpha diversity as evaluated based on the number of species and the Shannon index for each sample group. **b** Principal coordinates (PCo) analysis based on Bray–Curtis distance revealing the beta diversity in MCS patients and HC

We analyzed the number of species (≥ 0.1 relative abundance) and the Shannon index estimated from the mapping of the metagenomic reads to the reference genomes. The results revealed no statistical difference between the MCS and HC groups using Mann–Whitney U tests (Fig. 1a), suggesting that the alpha diversity of the gut microbiota was similar between the two groups. We then compared the beta diversity of the gut microbiota based on the Bray–Curtis distance/dissimilarity at the genus level. The results also showed no significant difference in the beta diversity of the gut microbiota between MCS patients and HC (PERMANOVA, R^2^ = 0.025, *p* = 0.227; Fig. 1b). Overall, we observed no substantial difference in alpha and beta diversity of the gut microbiota between the MCS patients and HC.

### MCS-associated microbiota alterations at the phylum/genus/species levels

**Fig. 2:**
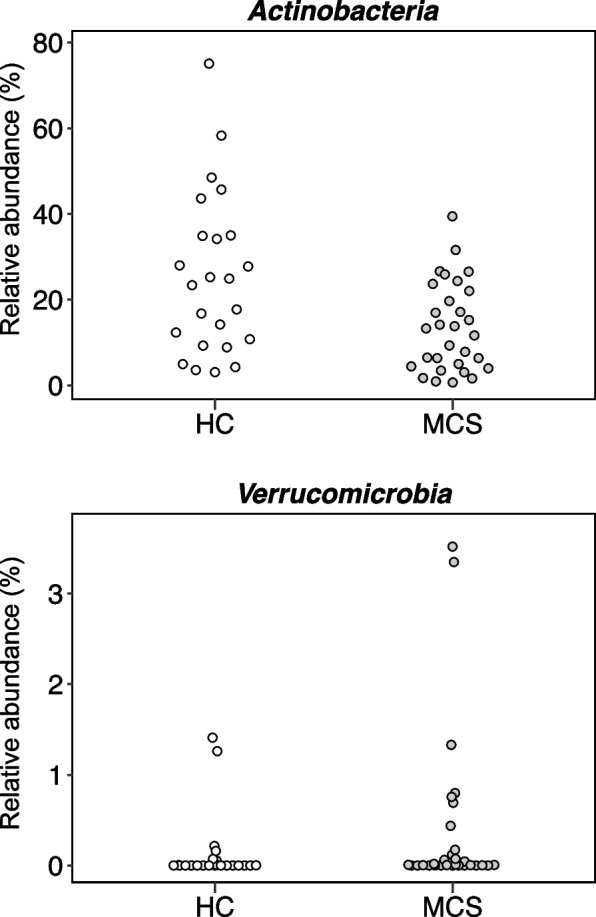
Relative abundances of significantly different phyla. Relative abundances of phyla that differed significantly between multiple chemical sensitivity (MCS, *n* = 30) patients and healthy controls (HC, *n* = 24)

**Fig. 3:**
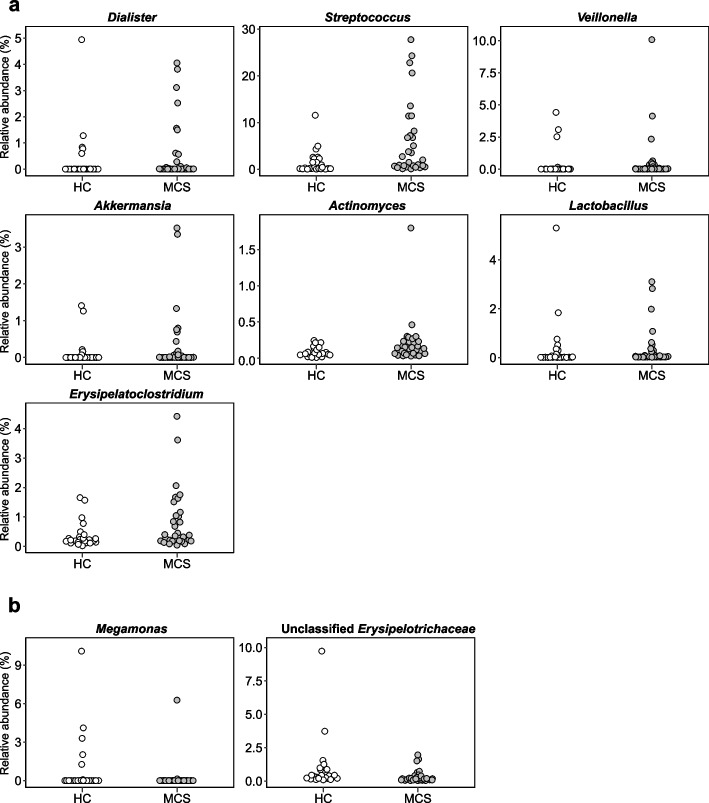
Relative abundances of significantly different genera. Relative abundances of genera that differed significantly between multiple chemical sensitivity (MCS, *n* = 30) patients and healthy controls (HC, *n* = 24). **a** Seven genera were enriched in MCS. **b** Two genera were depleted in MCS

**Fig. 4:**
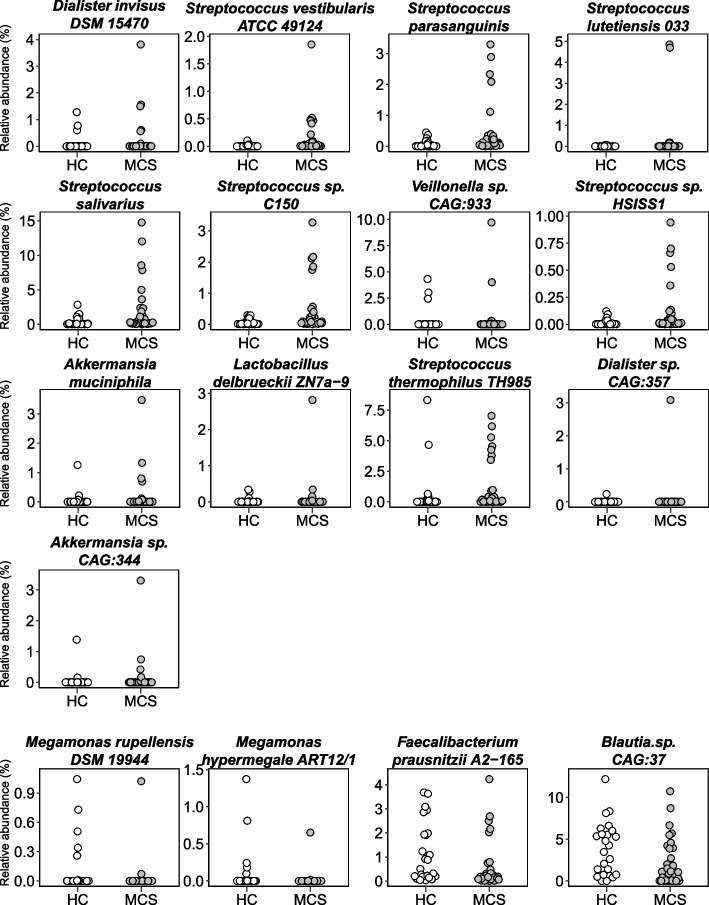
Relative abundances of significantly different species. Relative abundances of species that differed significantly between multiple chemical sensitivity (MCS, *n* = 30) patients and healthy controls (HC, *n* = 24) with a focus on the species discussed in this paper. **a** Species enriched in MCS. **b** Species depleted in MCS

We next explored bacterial taxa showing a significant change in relative abundance between MCS patients and HC. At the phylum level, *Actinobacteria* were significantly decreased in abundance (*p* < 0.01), whereas *Verrucomicrobia* were significantly increased in abundance (*p* < 0.01) in MCS compared with HC samples (Fig. 2). We further explored genera showing significant changes in relative abundance (≥ 0.1% average relative abundance) in MCS patients compared with HC. Seven genera, including *Dialister*, *Streptococcus*, *Veillonella*, *Akkermansia*, *Actinomyces*, *Lactobacillus*, and *Erysipelatoclostridium*, were more abundant, whereas *Meganomonas* and Unclassified *Erysipelotrichaceae* were less abundant in MCS patients than HC (Fig. 3). Species-level analysis identified 44 species, including *Akkermansia muciniphila*, *Faecalibacterium prausnitzii*, and *Streptococcus thermophilus,* showing significantly altered relative abundance between the two groups (≥ 0.1% average relative abundance; Fig. 4 and Figure S1 in Additional file 1), which included species belonging to the genera showing significant changes in relative abundance.

### Functional characterization of the MCS gut microbiota based on metagenomic data

**Fig. 5:**
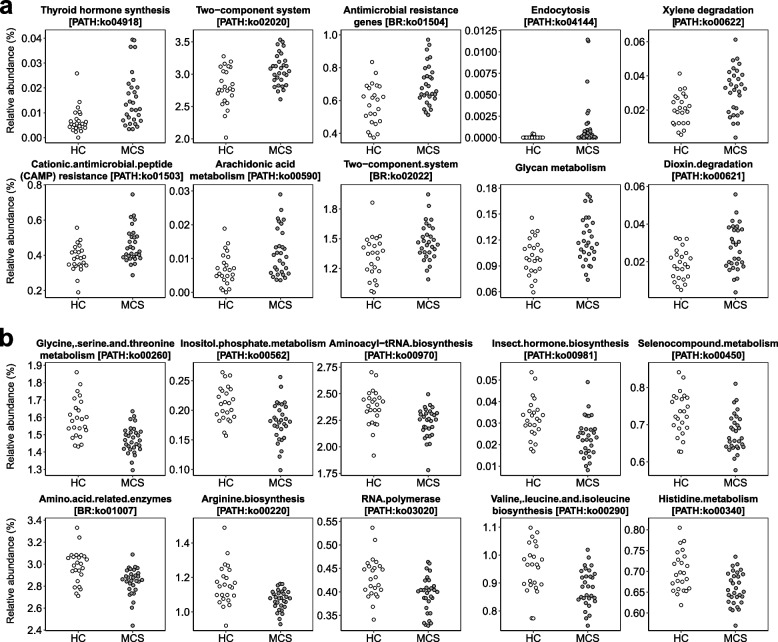
Relative abundances of top 10 significantly different functional pathways ranked by *p*-value. Relative abundances of top 10 functional pathways that significantly differed between multiple chemical sensitivity (MCS, *n* = 30) patients and healthy controls (HC, *n* = 24). **a** Pathways enriched in MCS. **b** Pathways depleted in MCS

Metagenomic reads were mapped to genes to characterize the gut microbiota functions that were significantly altered by MCS. Based on KEGG database analysis, we identified a total of 5,928 KOs in the metagenomic data of the two groups. In the KEGG analysis, all 5,928 detected KOs were used to aggregate pathways prior to statistical analysis. Among them, 567 KOs showed a significant difference in abundance between the two groups, including 301 KOs significantly enriched and 266 KOs significantly depleted in MSC (Mann–Whitney U test,* p* < 0.05). The top 10 KOs significantly enriched and depleted in MCS ranked by *p*-value are shown in Figure S2 (see Additional file 1). Analysis of KEGG level II functional categories based on the KOs revealed that six categories were significantly enriched (including drug resistance and signal transduction) and 10 significantly depleted (including amino acid metabolism, endocrine and metabolic disease, and nervous system) in MCS compared with HC (*p* < 0.05; Figure S3, in Additional file 1). Pathway analysis based on the KOs revealed that 74 pathways were significantly altered in MCS (*p* < 0.05), including 26 enriched and 48 depleted pathways (Tables 2 and 3). Among the top 10 pathways with a significant difference ranked by *p*-value, xylene and dioxin degradation pathways [PATH:ko00622 and ko00621] and pathways related to antimicrobial resistance, including the two-component system [PATH:ko02020], antimicrobial resistance genes [BR:ko01504], and cationic antimicrobial peptide resistance [PATH:ko01503], were significantly enriched in MCS compared with HC. Pathways involved in amino acid metabolism and amino acid synthesis, including glycine, serine, and threonine metabolism [PATH:ko00260], amino acid-related enzymes [BR:ko01007], and arginine biosynthesis [PATH:ko00220], were significantly depleted in MCS compared with HC (Fig. 5).

**Table 2:** Pathways significantly enriched in MCS

Definition	Pathway ID	*p*-value
Thyroid hormone synthesis	ko04918	0.00023872
Two-component system	ko02020	0.00123756
Antimicrobial resistance genes	ko01504	0.0015079
Endocytosis	ko04144	0.00151049
Xylene degradation	ko00622	0.00195105
Cationic antimicrobial peptide resistance	ko01503	0.00406715
Arachidonic acid metabolism	ko00590	0.00543367
Two-component system	ko02022	0.00719206
Glycan metabolism		0.00847242
Dioxin degradation	ko00621	0.01358711
MicroRNAs in cancer	ko05206	0.01358711
Huntington disease	ko05016	0.01660993
Chromosome and associated proteins	ko03036	0.02019539
Steroid biosynthesis	ko00100	0.02218133
Prokaryotic defense system	ko02048	0.02222404
Cholesterol metabolism	ko04979	0.02330197
Human T-cell leukemia virus 1 infection	ko05166	0.02330197
Neuroactive ligand-receptor interaction	ko04080	0.02330197
beta-Lactam resistance	ko01501	0.02330197
Glycosyltransferases	ko01003	0.03216668
Sulfur metabolism	ko00920	0.03363934
Protein kinases	ko01001	0.03363934
Cardiac muscle contraction	ko04260	0.04494874
Non-alcoholic fatty liver disease	ko04932	0.04494874
Parkinson disease	ko05012	0.04494874
Mannose type O-glycan biosynthesis	ko00515	0.04758114

*MCS* multiple chemical sensitivity, *HC* healthy controls

**Table 3:** Pathways significantly depleted in MCS

Definition	Pathway ID	*p*-value
Glycine, serine and threonine metabolism	ko00260	0.00003892
Inositol phosphate metabolism	ko00562	0.0005803
Aminoacyl-tRNA biosynthesis	ko00970	0.00062303
Insect hormone biosynthesis	ko00981	0.00071718
Selenocompound metabolism	ko00450	0.00132236
Amino acid related enzymes	ko01007	0.00160922
Arginine biosynthesis	ko00220	0.00171663
RNA polymerase	ko03020	0.00183047
Valine, leucine and isoleucine biosynthesis	ko00290	0.00195105
Histidine metabolism	ko00340	0.00195105
C5-Branched dibasic acid metabolism	ko00660	0.00361257
Vitamin B6 metabolism	ko00750	0.00383386
Global maps only		0.00457197
Thiamine metabolism	ko00730	0.00680488
Cysteine and methionine metabolism	ko00270	0.00994852
Autophagy—yeast	ko04138	0.01048819
Exosome	ko04147	0.01291053
Nitrogen metabolism	ko00910	0.01291053
Prenyltransferases	ko01006	0.01291053
Ubiquitin system	ko04121	0.01293742
Ribosome	ko03011	0.01429422
Ribosome	ko03010	0.01429422
Terpenoid backbone biosynthesis	ko00900	0.01660993
Steroid degradation	ko00984	0.01661341
Isoquinoline alkaloid biosynthesis	ko00950	0.01745052
Purine metabolism	ko00230	0.02019539
One carbon pool by folate	ko00670	0.02019539
Carbon fixation in photosynthetic organisms	ko00710	0.02019539
GABAergic synapse	ko04727	0.02019539
Glutamatergic synapse	ko04724	0.02222404
Translation factors	ko03012	0.02330197
Messenger RNA biogenesis	ko03019	0.02330197
Protein processing		0.02442413
Arabinogalactan biosynthesis—*Mycobacterium*	ko00572	0.02442413
Valine, leucine, and isoleucine degradation	ko00280	0.02807024
Cofactor metabolism		0.02807024
Pantothenate and CoA biosynthesis	ko00770	0.03074865
Cell cycle—*Caulobacter*	ko04112	0.03074865
Tryptophan metabolism	ko00380	0.03216668
Proteasome	ko03051	0.03363934
Novobiocin biosynthesis	ko00401	0.03516819
Glyoxylate and dicarboxylate metabolism	ko00630	0.03675485
D-Arginine and D-ornithine metabolism	ko00472	0.03675485
Fatty acid degradation	ko00071	0.03840094
Tropane, piperidine and pyridine alkaloid biosynthesis	ko00960	0.04010811
Propanoate metabolism	ko00640	0.04187801
Insulin resistance	ko04931	0.04187801
Proteoglycans in cancer	ko05205	0.04758114

*MCS* multiple chemical sensitivity, *HC* healthy controls

## Discussion

We conducted a metagenomic analysis to identify differences in the gut microbiota of patients with MCS based on gut microbiome sequencing data from 30 Japanese female patients with MCS and 24 age- and sex-matched HC. The MCS patients showed no less gut microbiome diversity than the HC; however, they exhibited significant differences in the bacterial abundance at the phylum, genus, and species levels compared with the HC. Furthermore, the MCS patients showed significantly different microbiota.

At the phylum level, *Verrucomicrobia* was significantly more abundant in MCS patients than in the HC. *Verrucomicrobia* includes *Akkermansia* [29], and we consider the significant abundance of *Verrucomicrobia* to be a result of increased *Akkermansia.*

At the genus level, *Akkermansia*, *Streptococcus*, *Dialister*, *Lactobacillus*, and *Veillonella* were more abundant in MCS patients than in HC. *Akkermancia* has anti-diabetic effects [30] and has been touted as the next generation of beneficial bacteria [31]; however, in a rat model of maternal separation stress, *Akkermansia* was increased in the early stage of stress, and the increase in *Akkermansia* was correlated with behavioral disorders [32]. Moreover, *Akkermansia* has a negative effect on the intestinal tract when timed with antimicrobial agents [33]. When dietary fiber is deficient, *A. muciniphila* feeds on mucous glycoproteins secreted by the host, resulting in marked decreases in the mucous layer and intestinal barrier function [34]. The disruption of the intestinal barrier function may cause food intolerances in MCS patients. A diet rich in unsaturated fatty acids and oral PPIs lead to an increase in *Streptococcus* [35, 36]. *Streptococcus* is associated with the worsening of functional dyspepsia symptoms, such as postprandial bloating and postprandial epigastric pain [37]. Therefore, *Streptococcus* may be involved in the gastrointestinal symptoms of MCS patients. *Dialister* is significantly less abundant in the microbiota of people living in rural areas than in those living in urban areas [38], which may explain why MCS is more common in developed countries than in developing countries. *Lactobacillus* and *Veillonella* are increased in Japanese patients with irritable bowel syndrome [39]. As Japanese people generally have low levels of lactose-degrading enzymes, lactose readily reaches the large intestine, where many *Lactobacillus* species reside [40]. Therefore, the increase in *Lactobacillus* may be associated with gastrointestinal symptoms in MCS patients. In addition to *Lactobacillus*, *Veillonella* are reported to generate acetic and propionic acid. High concentrations of acetic and propionic acids may be related to abdominal manifestations [39].

At the species level, *F. prausnitzii* was significantly less abundant in the MCS microbiota than in that of HC. A previous study reported that a decrease in *F. prausnitzii* lead to a decrease in regulatory T cells due to a decrease in butyrate acidity, which triggers the activation of Th17 lymphocytes and causes tissue damage. This system occurs not only in the digestive tract, but also in brain tissue [41, 42]. Therefore, the decrease in *F. prausnitzii* may be associated with the brain inflammation often observed in MCS. A low-fat diet increases the genera *Blautia* and *Faecalibacterium*, which are the key sources of energy for enterocytes and produce short-chain fatty acids with anti-inflammatory properties [43]. *Blautia sp*. were significantly lower in the MCS group than in the HC group, suggesting that the anti-inflammatory effect on the intestines may be reduced in these patients. In a Japanese study, people with a small visceral fat area had a high *Blautia* occupancy rate in the microbiota [44]. Furthermore, visceral fat has been associated with an abnormal brain network structure and an increased risk of cognitive decline [45]. In the future, it will be necessary to examine the visceral fat area and cognitive function of patients with MCS. *S. thermophilus* and *Streptococcus salivarius* were significantly more abundant in MCS patients than in HC. *S. thermophilus* are lactic acid bacteria that produce a large amount of lactic acid from lactose in milk. *S. thermophilus* is closely related to *S. salivarius*. Lactose intolerance and difficulty in digesting milk components in MCS patients may be associated with the increase in these bacterial species.

Based on gene and pathway analyses, we successfully elucidated novel functional aspects of the MCS gut metagenome. Xylene and dioxin degradation pathways were enriched in MCS. Volatile aromatic hydrocarbons such as benzene, toluene, ethylbenzene, and xylene are highly toxic and easily diffuse into the environment because of their volatility and water solubility. Shrimp (*Litopenaeus vannamei*) fed cottonseed protein concentrate had significantly fewer pathways for dioxin and xylene degradation than shrimp fed fish meal [46]. Although it is not clear whether MCS patients are more exposed or susceptible to xylenes or dioxins, the objective finding of an association between the gut microbiota and volatile aromatic hydrocarbons is important for dietary guidance for patients with MCS.

Pathways related to antimicrobial resistance were significantly enriched in MCS patients compared with HC. Bacteria have various signaling mechanisms, including the two-component system, that are not present in humans and allow bacteria to respond quickly to environmental changes [47]. The two-component system is also involved in antimicrobial resistance [48]. In this study, patients using antimicrobials or PPIs within the last six months were excluded. A gut microbiota altered by PPI use can be restored by discontinuing PPI use for two weeks [49]. Recent studies have indicated that the abundance of antibiotics resistance genes in the microbiome is positively correlated not only with the use of antimicrobial agents, but also with that of certain non-antimicrobial agents [50, 51]. We were unable to assess the relationship between non-antimicrobial agent use and increased bacterial resistance.

Pathways involved in amino acid metabolism and synthesis were depleted in MCS patients compared with HC. All of the 20 α-amino acids that make up proteins except glycine have mirror isomers termed l-amino and d-amino acids. With few exceptions, proteins are composed of l-amino acids, and only trace amounts of d-amino acids are detected in numerous organisms. However, bacteria produce a wide variety of d-amino acids, which have been shown to affect their hosts, including humans. Metabolism of d-amino acids derived from intestinal bacteria regulates host intestinal immunity [52, 53]. Further investigation of the function of amino acid metabolism in the gut microbiota is warranted.

This study has some limitations. First, the small sample size, single center, and the fact that the study was limited to Japanese women make it difficult to generalize the results of this study. However, as we eliminated ethnicity- and sex-related differences in the gut microbiota, we could focus on disease-specific microbiota changes. Second, the numbers of cases and controls differed because we used published data as a control group, selecting for age- and sex-matched subjects. Third, since there are no objective diagnostic criteria and biomarkers for MCS, disease uniformity could not be ensured. However, considering that the results of this study may contribute to the development of objective diagnostic criteria and treatment development, we carried out the study using globally accepted diagnostic criteria for MCS. Fourth, as we did not conduct a systematic survey of the patients’ diets, we were unable to analyze the relationship between diet and the microbiota. Fifth, the absolute number of gut microbiota was not evaluated, and the HC and MCS groups were compared in terms of relative abundance. Finally, due to the cross-sectional nature of the study, we cannot show a causal relationship between the onset of chemical sensitivity and changes in the gut microbiome.

In conclusion, the gut microbiota of patients with MCS shows dysbiosis and different bacterial functions related to exogenous chemicals and amino acid metabolism and synthesis. These findings may contribute to the further development of treatment for MCS.

### Supplementary Information

**Additional file 1. **Supplementary text and figures.

## Data Availability

All fecal metagenomic data were deposited in DNA Data Bank of Japan (DDBJ) with accession number DRA016817. https://ddbj.nig.ac.jp/search (Direct web link) https://ddbj.nig.ac.jp/search/en?query=%22DRA016817%22.

## Abbreviations

HC: Healthy control
KEGG: Kyoto Encyclopedia of Genes and Genomes
KOs: Kyoto Encyclopedia of Genes and Genomes orthologies
MCS: Multiple chemical sensitivity
PERMANOVA: Permutational multivariate analysis of variance
PPI: Proton pump inhibitor
QEESI: Quick Environmental Exposure and Sensitivity Inventory
