# Supplementary material for: Metagenomic gut microbiome analysis of Japanese patients with multiple chemical sensitivity/idiopathic environmental intolerance
Source: BMC Microbiol. 2024 Mar 11;24:84. doi: 10.1186/s12866-024-03239-y (PMC10926566; doi:10.1186/s12866-024-03239-y)
Supplement: Supplementary file 1 — Additional file 1. Supplementary text and figures. [file 12866_2024_3239_MOESM1_ESM.docx]

**Supplementary Materials**

**The Quick Environmental Exposure and Sensitivity Inventory (QEESI)**

The QEESI is a validated questionnaire to assess chemical intolerance in adults (Miller CS et al. Toxicol Ind Health 1999;15: 370-85 and 386-97). The QEESI consists of self-rating scales for symptoms, as well as responses to chemical and other common exposures, including foods, skin contactants, alcoholic beverages, and caffeine. Each of these three scales (Symptoms, Chemical Exposures, and Other Exposures) contains 10 items that participants rate from 0 to 10 in terms of the severity of their responses. The development, validation, sensitivity, and specificity of these scales have been published elsewhere.

*Chemical Exposures Scale (Q1)*

This scale asks participants about the severity of their responses to 10 common structurally diverse inhalants, rated from 0 to 10, as described above. Items include diesel or gas engine exhaust, tobacco smoke, insecticides, gasoline vapors, paint/paint thinner, fragrances, cleaning products, fresh tar or asphalt, nail polish/nail polish remover, hairspray, and new furnishings.

*Symptoms Scale (Q3)*

The Symptoms scale includes head-related, musculoskeletal, respiratory/mucus membrane, heart/chest, neuromuscular, gastrointestinal, cognitive, affective, skin, and genitourinary symptoms. Scoring for this scale is accomplished by asking participants to rate each item from 0 to 10 in a manner that best corresponds with the severity of their symptoms and responses to various substances: 0 = not a problem, 5 = moderate, 10 = severe or disabling. Scores on the 10 items for each scale are summed to obtain a total scale score (0–100).

*Other Exposures Scale*

The Other Exposures scale includes 10 questions concerning the severity of the subjects’ responses to a variety of other common exposures (non-inhalants), again asking them to rate their response severity from 0 to 10. These items include chlorinated tap water, foods/food additives, unusual cravings or feeling ill if a meal is missed, feeling ill after meals, caffeine, caffeine withdrawal, small amounts of alcoholic beverages, skin contactants, medical drugs/devices, and allergens (causing classic allergic responses of asthma, nasal symptoms, hives, eczema, or anaphylaxis).





Figure S1. Relative abundances of species other than those shown in Figure 4 that differed significantly between multiple chemical sensitivity (MCS, n = 30) patients and healthy controls (HC, n = 24). (a) Species enriched in MCS. (b) Species depleted in MCS.





Figure S2. Top 10 KEGG Orthologies (KOs) that differed significantly between multiple chemical sensitivity (MCS, n = 30) patients and healthy controls (HC, n = 24) ranked by p-value. (a) KOs enriched in MCS. (b) KOs depleted in MCS.


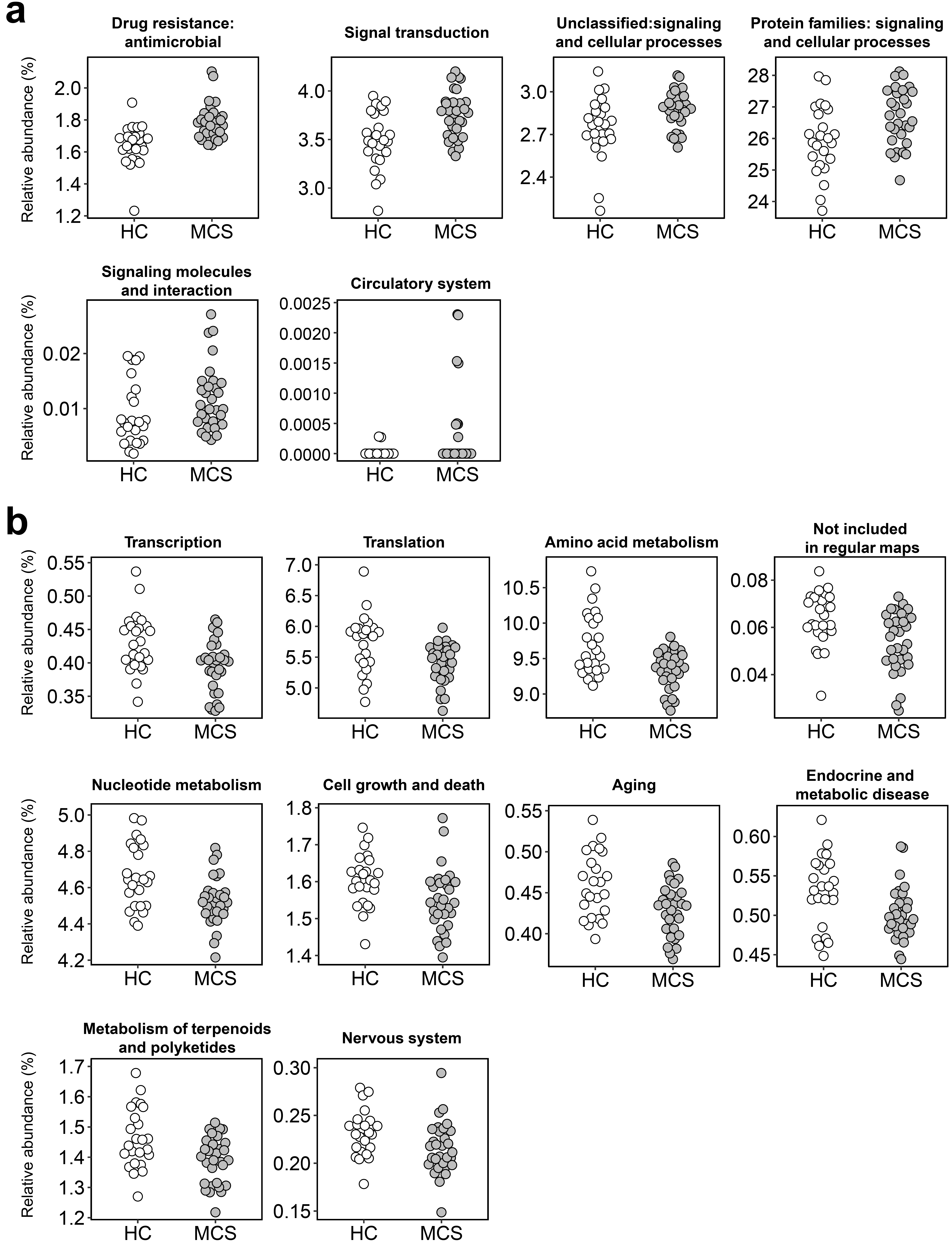


Figure S3. KEGG level II functional categories that differed significantly between multiple chemical sensitivity (MCS, n = 30) patients and healthy controls (HC, n = 24) based on KEGG Orthologies (KOs). (a) Categories enriched in MCS. (b) Categories depleted in MCS.
